# Cataract surgery outcomes in pseudoexfoliation syndrome: a large multicenter database study

**DOI:** 10.3389/fopht.2026.1687620

**Published:** 2026-02-12

**Authors:** Ahmed F. Shakarchi, Mohamed K. Soliman, Fatma F. Shakarchi, Abdelrahman M. Elhusseiny, Yit C. Yang, Ahmed B. Sallam

**Affiliations:** 1 Department of Ophthalmology, Harvey and Bernice Jones Eye Institute, University of Arkansas for Medical Sciences, Little Rock, AR, United States; 2 Department of Ophthalmology, Ohio State University, Columbus, OH, United States; 3 International Committee of the Red Cross (ICRC), Geneva, Switzerland; 4 Wolverhampton Eye Infirmary, Royal Wolverhampton Hospitals National Health Service (NHS) Trust, New Cross, Wolverhampton, United Kingdom; 5 Department of Ophthalmology, Ain Shams University Hospitals, Cairo, Egypt

**Keywords:** cataract surgery, PEX, phacoemulsification, pseudoexfoliation syndrome, surgical outcomes

## Abstract

**Aim:**

To evaluate intraoperative and postoperative complications and visual acuity (VA) outcomes of cataract surgery in pseudoexfoliation (PEX) eyes compared to non-PEX eyes.

**Methods:**

We included eyes undergoing phacoemulsification across eight centers in the United Kingdom between July 2003 and March 2015. We categorized these into four groups based on the presence of PEX and other ocular comorbidities. Complications and VA were calculated among each group. Multivariable adjusted models were used to obtain adjusted differences in VA outcomes and adjusted relative risks (RR) of complications.

**Results:**

172,008 eyes were included. PEX was present in 1.2% of the study population. PEX eyes had more posterior capsular rupture, zonular dialysis, nucleus drop, and conversion to extracapsular extraction (adjusted RR = 2.3 [95% CI = 1.7 – 3.0], 6.7 [4.9 – 9.0], 2.5 [1.2 – 5.4], and 2.6 [1.03 – 6.3], respectively). Postoperatively, PEX eyes experienced more intraocular pressure ≥ 22 mmHg and IOL dislocation requiring surgical intervention (adjusted RR = 1.6 [1.1 – 2.3] and 7.3 [4.4 – 12.2], respectively). After accounting for the effect of ocular copathology, PEX eyes had similar postoperative VA outcomes (adjusted RR of achieving LogMAR <= 0.3 = 0.99, 95% CI = 0.95 – 1.02) and VA gain ≥ 0.3 logMAR (adjusted RR = 1.05, 95% CI = 0.99 – 1.11) at 4–12 weeks, compared to non-PEX eyes.

**Conclusions:**

Cataract surgery in PEX has more intraoperative complications, but the visual outcomes were favorable. These findings can inform clinical decision-making and patient counseling in managing cataracts in individuals with PEX.

## Introduction

Pseudoexfoliation (PEX) is a systemic disease particularly common in older individuals of Scandinavian or Mediterranean heritage. It is characterized by the deposition of fibrillary proteinaceous material in various body tissues (1). Most of these depositions are not of clinical significance, other than a potentially increased risk of cardiovascular disease (2). In the eye, however, PEX is associated with glaucoma, poor pupil dilation, zonular weakness and dislocation or subluxation of the crystalline lens (3).

Cataract surgery in eyes with PEX has been associated with an increased risk of intraoperative zonular dialysis, posterior capsular tear, lens dislocation and vitreous prolapse (4–6). Postoperatively, patients have been found to have an increased risk of inflammation, elevated intraocular pressure and IOL dislocation (3, 7–9). While most studies agree that there is an increased risk of these complications in cataract surgery in PEX, there are inconsistencies in the literature (7). For instance, two studies from Malaysia had conflicting results in terms of increased risk of posterior capsule rupture (PCR) in eyes with PEX. Thanigasalam et al. found no increased risk of PCR (10), whereas Salowi et al. showed an increased risk in their cohort (5). Similarly, for vitreous loss during surgery, Hyams et al. found no increased risk (6), while Zare et al. found higher risk of vitreous loss in pseudoexfoliation (11). Currently, there are no large studies to provide reliable figures for these complications in association with PEX.

With regards to visual outcomes after cataract surgery, the results are also mixed. A study from Finland found less visual gains and worse visual acuity (VA) outcomes in pseudoexfoliation (12), whereas a large study from the Aravind Eye Institute in India found VA outcomes equivalent to controls in uncomplicated pseudoexfoliation (13).

In this study, we utilize a large clinical dataset to evaluate the intraoperative and postoperative complications and visual outcomes of cataract surgery in eyes with and without PEX.

## Methods

### Data

We collected anonymized cataract surgery data from eight National Health Service (NHS) centers in the United Kingdom, covering a 12-year period (July 2003–March 2015). Each site had a large ophthalmology department with a representative patient mix and used the same electronic medical record (EMR) system (Medisoft Ophthalmology; Medisoft Limited). The data were combined into a single database.

Details of care standards and data extraction at these centers have been described previously (14). The study adhered to the principles of the Declaration of Helsinki. Because the data were de-identified, the University of Arkansas for Medical Sciences Institutional Review Board exempted it from review.

### Study population

We included all eyes that underwent phacoemulsification cataract surgery, including cases converted intraoperatively to extracapsular extraction. Exclusions were eyes with planned intracapsular or non-phacoemulsification extracapsular surgery, as well as eyes undergoing combined cataract and other ocular procedures (e.g., retinal or glaucoma surgery).

We accounted for the potential confounding effect of ocular comorbidities primarily through stratification into four categories based on the presence or absence of PEX and copathology, such as glaucoma, corneal disease, and retinal or optic nerve pathology. This design allowed comparison of eyes with PEX but without other disease to a clean reference group. Disease severity (e.g., glaucoma stage or degree of macular change) was not available in the dataset. The four groups were:

1. Eyes without PEX and without other ocular comorbidities (reference group: -ve PEX -ve copath).
2. Eyes with PEX but no other ocular comorbidities (+ve PEX -ve copath).
3. Eyes without PEX but with other ocular comorbidities (-ve PEX +ve copath).
4. Eyes with both PEX and other ocular comorbidities (+ve PEX +ve copath).

### Outcome measures

We compared the four groups with respect to preoperative, intraoperative, and postoperative variables. Preoperative data included demographics, diabetes status, presence of advanced white or brunescent cataract, pupil size, and ocular comorbidities. The ocular conditions assessed were age-related macular degeneration (ARMD), amblyopia, corneal pathologies, diabetic retinopathy or macular edema, epiretinal membrane, glaucoma, high myopia, optic nerve disease, prior vitrectomy, previous trabeculectomy, retinal vein occlusion, and uveitis.

Intraoperative outcomes included the use of pupil expansion devices (e.g., rings or iris hooks), capsular tension rings (CTR), conversion to extracapsular extraction, PCR, dropped nucleus, zonular dialysis (with or without vitreous loss), and iris trauma. Complication reporting in the EMR was mandatory: surgeons had to indicate the presence or absence of intraoperative complications before finalizing their operative note, selecting from a standardized list of recognized cataract surgery complications.

Postoperative outcomes included clinically significant cystoid macular edema (CME), intraocular lens (IOL) dislocation requiring explantation, exchange, or repositioning, and intraocular pressure (IOP) spikes, defined as IOP >21 mmHg within three months after surgery. CME was defined by a clinical diagnosis within 90 days. Ancillary imaging (optical coherence tomography “OCT” or fluorescein angiography) was ordered at the surgeon’s discretion, usually for patients with poor visual recovery; therefore, CME captured here reflects clinically significant disease (15).

VA was assessed pre- and postoperatively, with changes in VA calculated accordingly. Snellen acuities were converted to logMAR. Owing to the retrospective design, postoperative VA was reported as either best uncorrected or corrected distance acuity. VA outcomes were recorded across three intervals: 0–4 weeks, 4–12 weeks, and 12–24 weeks. The primary endpoints were VA between 4–12 weeks and the incidence of intra- and postoperative complications, with particular focus on comparing the +PEX/–copath group against the reference group.

### Statistical analysis

For each of the four study groups, categorical and continuous outcomes were compared using chi-square tests and analysis of variance, respectively. Adjusted relative risks (RRs) were calculated using Poisson regression models with robust error variance. Multivariable linear regression was applied to estimate adjusted differences. All analyses were conducted using R software, version 3.5.3 (R Foundation for Statistical Computing, Vienna, Austria; www.R-project.org).

## Results

### Demographics of the study eyes

**Figure 1:**
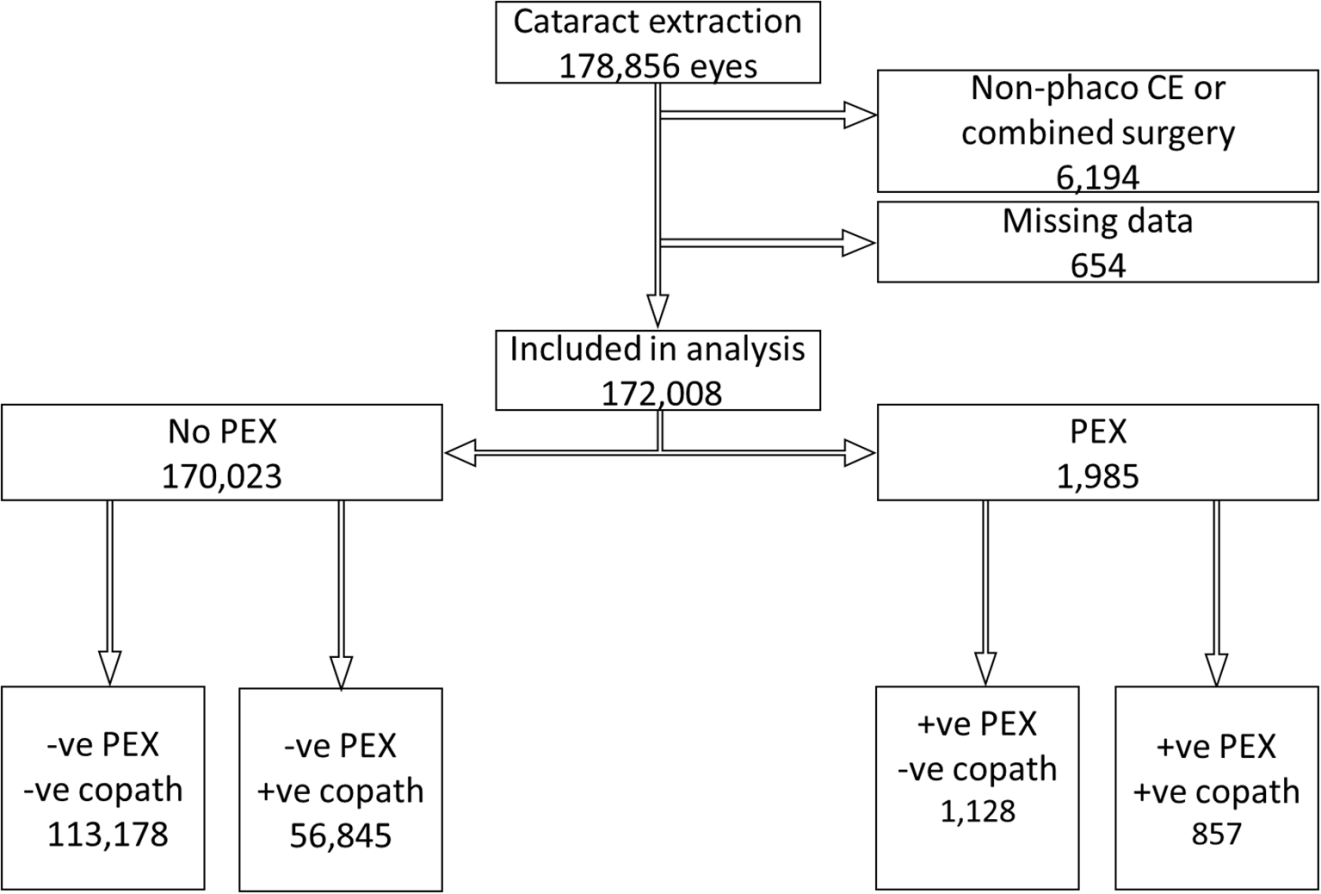
Flowchart of patients included in the study. CE, cataract extraction; PEX, pseudoexfoliation; copath, history of any of the following: age-related macular degeneration, amblyopia, corneal pathology, diabetic retinopathy or macular edema, epiretinal membrane, glaucoma, high myopia, optic nerve disease, previous vitrectomy, previous trabeculectomy, retinal vein occlusion, and uveitis.

In total, 172,008 eyes were eligible for analysis: 113,178 (65.8%) in the -ve PEX -ve copath group, 56,845 (33.0%) in -ve PEX +ve copath, 1,128 (0.7%) in +ve PEX -ve copath, and 857 (0.5%) in the +ve PEX +ve copath group (**Figure 1**). The mean age for the entire study population was 75.1 ± 10.6 years and patients with PEX were about 4 years older than those without (p < 0.001). Overall, females represented 59.6% of the study population and 17.5% were diabetics.

Eyes with PEX had more advanced cataracts and small pupils than non-PEX eyes (p < 0.001 for both). Advanced cataract represented 3.1% of the -ve PEX -ve copath group, 11.7% of eyes with +ve PEX -ve copath and 19.8% of eyes with +ve PEX +ve copath. Similarly, 2.4% of -ve PEX -ve copath eyes had small pupil, whereas 15.2% of +ve PEX -ve copath eyes and 20.8% of +ve PEX +ve copath eyes had small pupils. Eyes with PEX had more glaucoma and a history of trabeculectomy compared to eyes without PEX (p < 0.001 for both). On the other hand, PEX eyes had fewer ARMD, diabetic retinopathy or macular edema, high myopia, and a history of vitrectomy (p < 0.001 for all) (**Table 1**).

**Table 1:** Preoperative characteristics in eyes with and without pseudoexfoliation.

Variable	Overall N (%)	-ve PEX -ve copath N (%)	-ve PEX +ve copath N (%)	+ve PEX -ve copath N (%)	+ve PEX +ve copath N (%)	P-value^1^
N	172,008	113,178	56,845	1,128	857	
Age, mean ± SD	75.1 ± 10.6	75.0 ± 10.1	75.1 ± 11.4	79.0 ± 8.3	79.2 ± 9.3	< 0.001*
Female	102,491 (59.6)	68,453 (60.5)	32,773 (57.7)	750 (66.5)	515 (60.1)	< 0.001*
Diabetes mellitus	30,070 (17.5)	16,358 (14.5)	13,384 (23.5)	172 (15.2)	156 (18.2)	< 0.001*
White/brunescent cataract	6,235 (3.6)	3,490 (3.1)	2,443 (4.3)	132 (11.7)	170 (19.8)	< 0.001*
Small pupil	5,736 (3.3)	2,711 (2.4)	2,676 (4.7)	171 (15.2)	178 (20.8)	< 0.001*
Ocular pathologies						
ARMD	14,800 (8.6)		14,662 (25.8)		138 (16.1)	< 0.001*
Amblyopia	2,464 (1.4)		2,436 (4.3)		28 (3.3)	0.2
Corneal pathology	4,887 (2.8)		4,820 (8.5)		67 (7.8)	0.5
Diabetic retinopathy or DME	8,273 (4.8)		8,210 (14.4)		63 (7.4)	< 0.001*
ERM	1,227 (0.7)		1,216 (2.1)		11 (1.3)	0.1
Glaucoma	13,133 (7.6)		12,660 (22.3)		473 (55.2)	< 0.001*
High myopia	5,784 (3.4)		5,757 (10.1)		27 (3.2)	< 0.001*
Optic nerve/CNS disease	627 (0.4)		619 (1.1)		8 (0.9)	0.8
Previous PPV	3,361 (2.0)		3,343 (5.9)		18 (2.1)	< 0.001*
Previous Trabeculectomy	662 (0.4)		635 (1.1)		27 (3.2)	< 0.001*
RVO	1,605 (0.9)		1,580 (2.8)		25 (2.9)	0.9
Uveitis	1,908 (1.1)		1,877 (3.3)		31 (3.6)	0.7

PEX, pseudoexfoliation; copath, copathologies; ARMD, age-related macular degeneration; DME, diabetic macular edema; ERM, epiretinal membrane; PPV, pars plana vitrectomy; RVO, retinal vein occlusion.

*Statistically significant.

^1^analysis of variance or Chi square. For ocular pathologies, p-value is for Chi square test comparing +ve PEX +ve copath to -ve PEX +ve copath groups.

### Intraoperative complications

**Figure 2:**
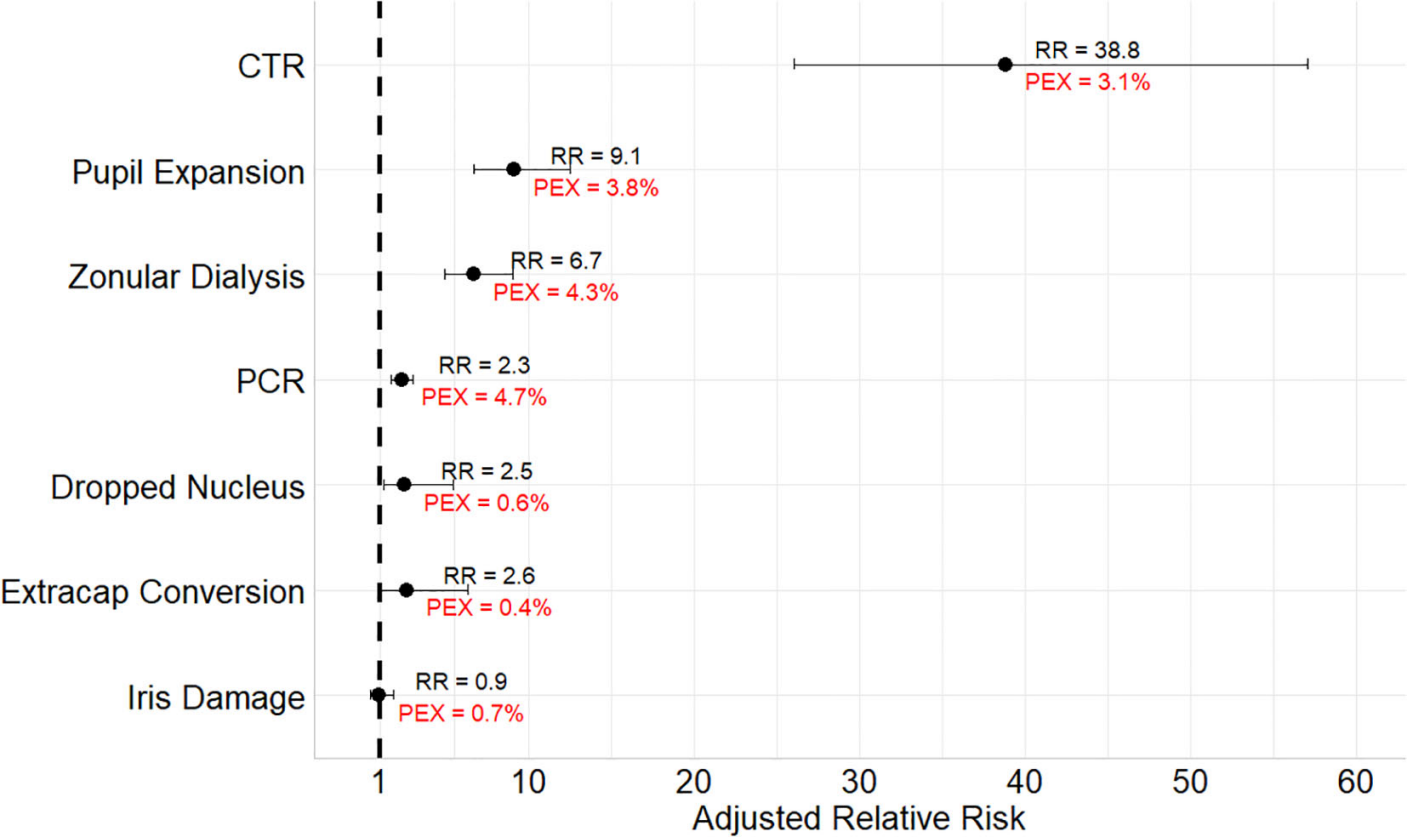
Relative risks of intraoperative complications in eyes with pseudoexfoliation compared to eyes without after excluding eyes with copathologies and after adjusting for confounders. PEX, percentage of the intraoperative complications in pseudoexfoliation eyes without copathologies; RR, relative risk; CTR, capsular tension ring; PCR, posterior capsule rupture. Models are adjusted for age, sex, diabetes status, small pupil, and advanced cataract. One exception is the model for pupil expansion device did not include small pupil as a covariate.

**Supplementary Table 1** lists the incidence of the intraoperative findings and complications in all 4 study groups. Compared to the reference group, cataract surgery in +ve PEX -ve copath eyes was associated with CTR use (RR = 38.8 [95% confidence interval (CI) = 26 – 57]), pupil expansion device use (RR = 9.1, 95% CI = 6.7 – 12.5), zonular dialysis (RR = 6.7, 95% CI = 4.9 – 9.0), conversion to extracapsular extraction (RR = 2.6, 95% CI = 1.03 – 6.3), dropped nucleus (RR = 2.5, 95% CI = 1.2 – 5.4) and PCR (RR = 2.3, 95% CI = 1.7 – 3.0) (**Figure 2**). Among eyes with +ve PEX and +ve copath, CTR use, pupil expansion and conversion to extracapsular extraction rates were even higher.

### Postoperative complications

**Figure 3:**
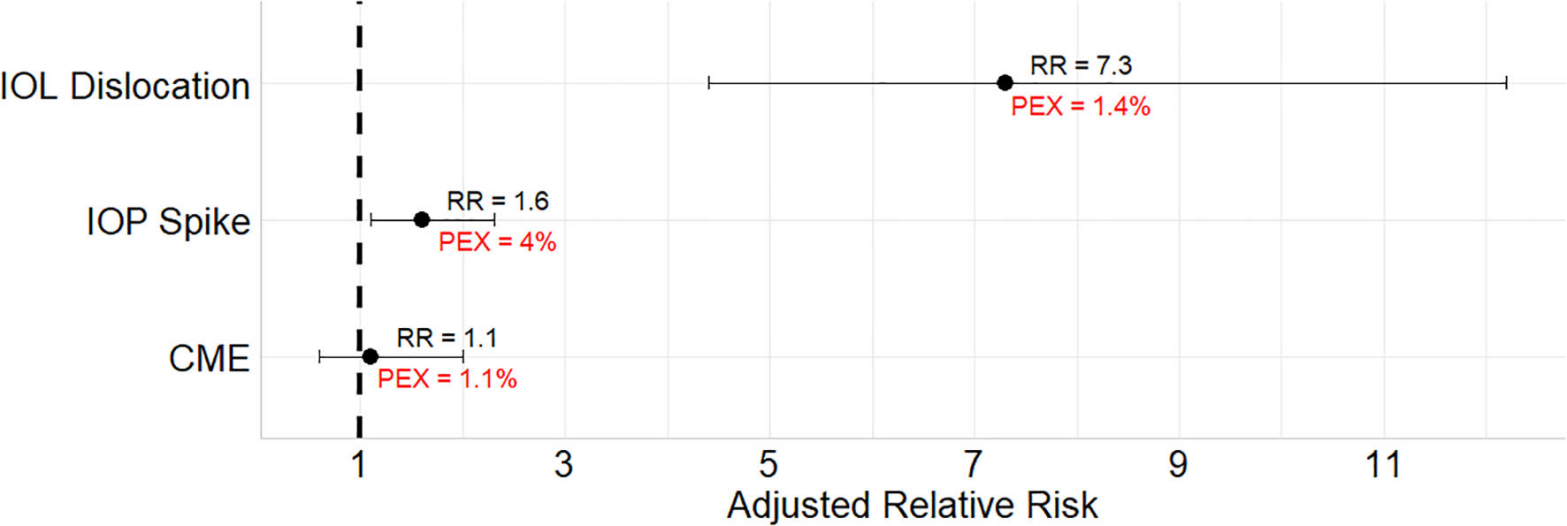
Relative risks of postoperative complications in eyes with pseudoexfoliation compared to eyes without after excluding eyes with copathologies and after adjusting for confounders. PEX, percentage of the postoperative complications in pseudoexfoliation eyes without copathologies; RR, relative risk; IOL, intraocular lens; IOP, intraocular pressure; CME, cystoid macular edema. Models are adjusted for age, sex, diabetes status, small pupil, and advanced cataract. IOP Spike is defined as first recorded postoperative IOP within 3 months > 21 mmHg.

**Supplementary Table 2** shows the incidence of postoperative complications in all 4 study groups. Compared to the reference group, +ve PEX -ve copath eyes experienced more IOL dislocations postoperatively requiring surgical correction (adjusted RR = 7.3, 91% CI = 4.4 – 12.2) and more IOP spikes (RR = 1.6, 95% CI = 1.1 – 2.3). These IOP spikes were documented in PEX eyes without diagnosed glaucoma. There was no difference in the risk of postoperative CME between PEX and non-PEX eyes. (See **Figure 3**) Among eyes with +ve PEX and +ve copath, these relative risks were even higher. There was no increased risk of having corneal transplantation or glaucoma surgery among eyes with PEX only.

### Visual acuity

**Figure 4:**
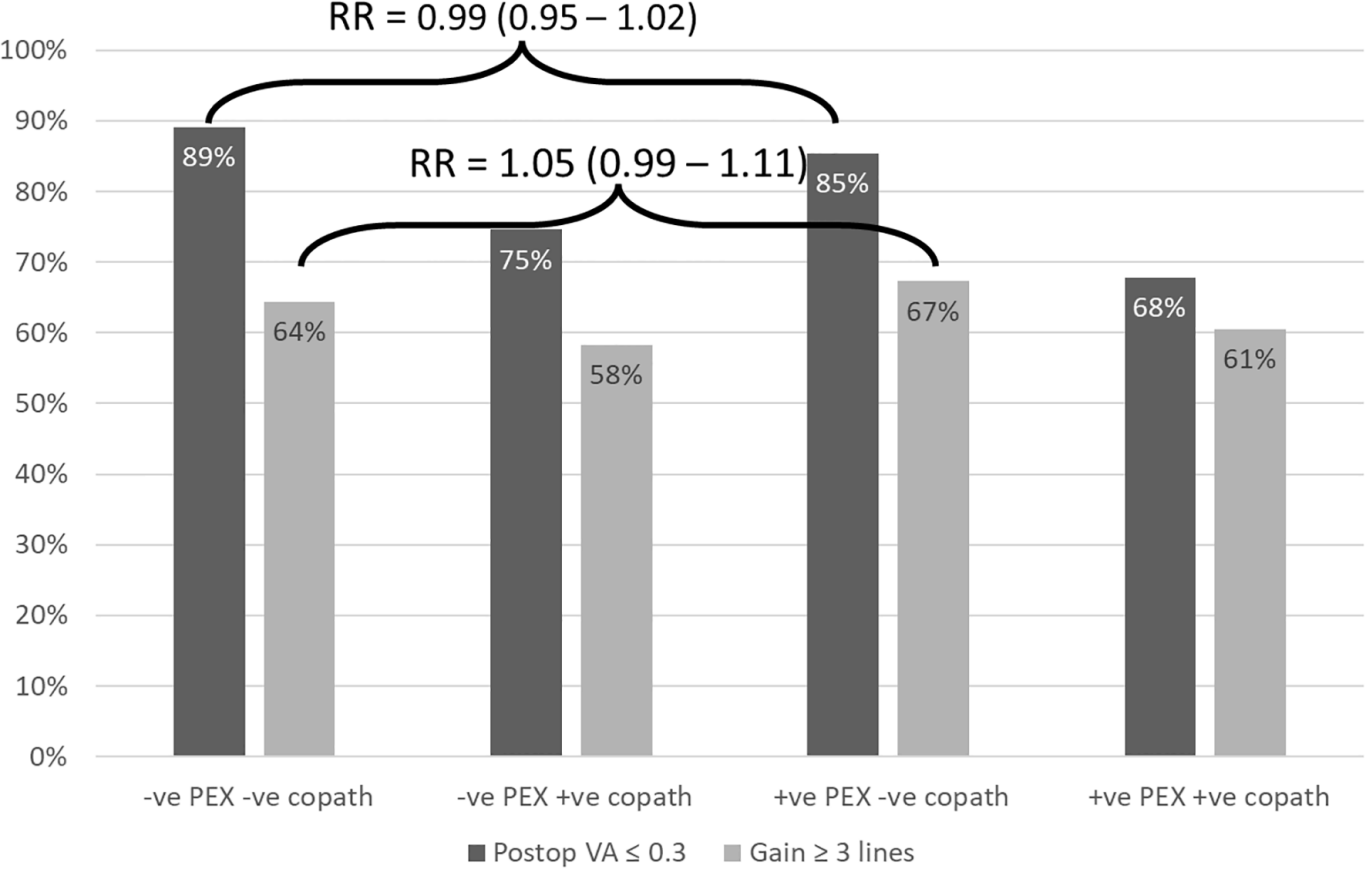
Postoperative visual acuity at 4–12 weeks greater than or equal to 20/40 (logMAR 0.3) and visual acuity gain of ≥3 lines (≥ 0.3 logMAR) in eyes with and without pseudoexfoliation and/or copathologies. RR, relative risk; VA, visual acuity; logMAR, logarithm of the minimum angle of resolution; PEX, pseudoexfoliation; copath, history of any of the following: age-related macular degeneration, amblyopia, corneal pathology, diabetic retinopathy or macular edema, epiretinal membrane, glaucoma, high myopia, optic nerve disease, previous vitrectomy, previous trabeculectomy, retinal vein occlusion, and uveitis.

**Supplementary Table 3** shows the preoperative and postoperative VA at various time points in all 4 study groups. Compared to the reference group, the +ve PEX -ve copath group had similar proportion of eyes with postoperative VA of ≥ 20/40 (logMAR ≤ 0.3), 85.4% *vs* 89.1%, at 4–12 weeks (adjusted RR = 0.99, 95% CI = 0.95 – 1.02). Also, the proportions of eyes achieving ≥ 3 lines of vision gain (≥ 0.3 logMAR), at the same time point, were similar (67.3% *vs* 64.4%, respectively) (adjusted RR = 1.05, 95% CI = 0.99 – 1.11) (**Figure 4**). However, eyes with +ve PEX and +ve copath were significantly less likely to achieve VA >= 20/40 compared to reference (RR = 0.79, 95% CI = 0.75 – 0.84).

## Discussion

In this large multicenter study of over 172,000 eyes undergoing cataract surgery, PEX was present in approximately 1.2% of the study population, which is consistent with previous studies from the UK (7, 16). We found a higher rate of intraoperative complications, including PCR, dropped nuclear fragments, and zonular dialysis, in eyes with PEX compared to eyes without. Postoperatively, eyes with PEX had a higher rate of IOP spike even without a preexisting glaucoma and more IOL dislocations requiring surgical intervention. In the absence of associated ocular pathology, eyes with PEX experienced similar visual acuity gains after cataract surgery compared to eyes without PEX.

Cataract surgery in PEX was more complex, requiring use of pupil expansion and capsular support devices. There were also higher rates of complications including zonular dialysis, PCR, dropped nucleus and conversion to extracapsular extraction. While there have been inconsistencies, most studies have linked higher PCR rates during cataract surgery to PEX (4–6, 17). In a single center UK study of 23,329 eyes (280 with PEX) eyes, PEX was not associated with PCR and the authors speculated that it may be because their analysis was adjusted for zonular dialysis that was not performed in other studies that showed positive association (7). However, in our analysis, the association between PEX and PCR during cataract surgery persisted even after adjusting for zonular dialysis. Therefore, we can conclude that PEX is an independent risk factor for PCR during cataract surgery. Furthermore, our study underscores that the presence of concurrent ocular pathology in PEX can exacerbate the occurrence of certain intraoperative events such as the use of CTR or pupil expansion devices and the rate of conversion to extracapsular extraction.

Fibrillary material deposition leads to zonular weakness and intraoperative or postoperative zonular dialysis (7, 18). There was nearly a 7-fold increase in zonular dialysis in eyes with PEX in our cohort and about a 39-fold increased use of CTRs. This higher need for CTRs may reflect surgeon preferences for prophylactic placement. Despite the frequent use of CTR, postoperative IOL dislocation occurred more frequently in PEX eyes. After adjusting for CTR use, the association between PEX and zonular dialysis decreased to a 4-fold increase, indicating a partial but not complete protective role of CTR against IOL dislocation.

Eyes with PEX had preoperative VA that was about a line worse than eyes without PEX after accounting for covariates including the presence of advanced cataract and excluding copathologies. Both PEX and non-PEX eyes had clinically significant gains of vision following cataract surgery. The proportion of eyes achieving postoperative VA of ≥20/40 or better was similar among eyes with and without PEX. Similarly, the proportion of eyes gaining ≥3 lines after surgery was also similar. Postoperative VA and VA gain were worse in the groups with copathologies, indicating the visual outcomes of cataract surgery in pseudoexfoliation may be mainly driven by copathologies rather than pseudoexfoliation itself. This may explain why some studies that did not stratify for copathologies found differences in VA outcomes between and PEX and non-PEX eyes (12, 19).

The limitations of this study include the retrospective design of clinical data. The retrospective nature means that there is inherent risk of bias and residual confounding in the analyses. However, this clinical dataset is more representative of the real-world setting. Therefore, the risks and outcomes shown here should be informative to clinicians and patients. The dataset lacks information on the severity of PEX, which could have impacted outcomes. On the other hand, the study has important strengths: While a prospective study represents a higher level of evidence, the execution of such a study is difficult given the rate of this disease. The large sample size and multicenter design of the current study increase the generalizability of the findings. The analyses were stratified by the presence of non-PEX ocular copathologies. This stratification accounted for the confounding effect of ocular comorbidities on the visual outcomes and intraoperative complications, revealing important insights on the drivers of postoperative outcomes in eyes with PEX.

This multicenter study defines and quantifies the visual outcome and the rate of perioperative complications of cataract surgery in eyes with PEX. The study highlights the challenges of cataract surgery in patients with PEX, such as increased risk of PCR, zonular dialysis and IOL dislocations. Despite the latter challenges, cataract surgery in eyes with PEX can result in VA gains that are equivalent to eyes without PEX. Ocular comorbidities are the main drivers of reduced visual acuity outcomes after cataract surgery in PEX. The results of this study may help to inform clinical decision-making and patient counseling.

## Data Availability

The data analyzed in this study is subject to the following licenses/restrictions: Data available upon request. Requests to access these datasets should be directed to ASallam@uams.edu.
