# Supplementary material for: Cataract surgery outcomes in pseudoexfoliation syndrome: a large multicenter database study
Source: Front Ophthalmol (Lausanne). 2026 Feb 12;6:1687620. doi: 10.3389/fopht.2026.1687620 (PMC12935656; doi:10.3389/fopht.2026.1687620)
Supplement: Supplementary file 1 [file DataSheet1.docx]

**Cataract Surgery Outcomes in Pseudoexfoliation Syndrome: A Multicenter Study Database Study**

**Supplemental Table 1**. The incidence and risk of intraoperative findings and complications in eyes with and without pseudoexfoliation

| **Variable** | **Overall (%)** |  | **-ve PEX -ve copath** |  | **-ve PEX +ve copath** |  | **+ve PEX -ve copath (%)** | |  | **+ve PEX +ve copath** | |
| --- | --- | --- | --- | --- | --- | --- | --- | --- | --- | --- | --- |
|  | **N (%)** |  | **N (%)** |  | **N (%)** |  | **N (%)** | **RR (95% CI)^1^** |  | **N (%)** | **RR (95% CI)^1^** |
| **N** | 172,008 |  | 113,178 |  | 56,845 |  | 1,128 |  |  | 857 |  |
| **CTR** | 410 (0.2) |  | 101 (0.1) |  | 239 (0.4) |  | 35 (3.1) | 38.8 (26 – 57)* |  | 35 (4.1) | 46.8 (31.0 – 70.7)* |
| **Pupil expansion device** | 1,268 (0.7) |  | 409 (0.4) |  | 764 (1.3) |  | 43 (3.8) | 9.1 (6.7 – 12.5)* |  | 52 (6.1) | 12.9 (9.6 – 17.2)* |
| **Zonular dialysis** | 1,073 (0.6) |  | 562 (0.5) |  | 420 (0.7) |  | 48 (4.3) | 6.7 (4.9 – 9.0)* |  | 43 (5) | 6.9 (5.0 – 9.5)* |
| **PCR** | 3,140 (1.8) |  | 1,869 (1.7) |  | 1,155 (2.0) |  | 53 (4.7) | 2.3 (1.7 – 3.0)*^2^ |  | 63 (7.4) | 2.2 (1.7 – 2.8)* |
| **Dropped nucleus** | 336 (0.2) |  | 186 (0.2) |  | 138 (0.2) |  | 7 (0.6) | 2.5 (1.2 – 5.4)* |  | 5 (0.6) | 1.9 (0.8 – 4.6) |
| **Conversion to ECCE** | 204 (0.1) |  | 115 (0.1) |  | 74 (0.1) |  | 5 (0.4) | 2.6 (1.03 – 6.3)* |  | 10 (1.2) | 4.8 (2.4 – 9.6)* |
| **Iris damage** | 744 (0.4) |  | 429 (0.4) |  | 300 (0.5) |  | 8 (0.7) | 0.9 (0.4 – 1.8) |  | 7 (0.8) | 0.8 (0.4 – 1.7) |

Abbreviations: PEX = pseudoexfoliation, copath = copathologies, RR = relative risk, CTR = capsular tension ring, PCR = posterior capsule rupture, ECCE = extracapsular cataract extraction

* Statistically significant

1 compared to -ve PEX -ve copath in a multivariable model adjusted for age, sex, diabetes status, small pupil, and advanced cataract. One exception is the model for pupil expansion device did not include small pupil as a covariate.

2 In an additional multivariable model adjusted for above factors as well as zonular dialysis, RR for +ve PEX -ve copath compared to -ve PEX -ve copath = 1.7, 95% CI = 1.3 – 2.2

**Supplemental Table 2**. The incidence and risk of postoperative complications in eyes with and without pseudoexfoliation

| **Variable** | **Overall (%)** |  | **-ve PEX -ve copath** |  | **-ve PEX +ve copath** |  | **+ve PEX -ve copath (%)** | |  | **+ve PEX +ve copath** | |
| --- | --- | --- | --- | --- | --- | --- | --- | --- | --- | --- | --- |
|  | **N (%)** |  | **N (%)** |  | **N (%)** |  | **N (%)** | **RR (95% CI)^1^** |  | **N (%)** | **RR (95% CI)^1^** |
| **N** | 172,008 |  | 113,178 |  | 56,845 |  | 1,128 |  |  | 857 |  |
| **IOL dislocation** | 404 (0.2) |  | 219 (0.2) |  | 151 (0.3) |  | 16 (1.4) | 7.3 (4.4 – 12.2)*^2^ |  | 18 (2.1) | 9.8 (6.0 – 16.3)* |
| **IOP spike^3^** | 3,252 (3.4) |  | 1,680 (2.6) |  | 1,505 (4.9) |  | 25 (4.0) | 1.6 (1.1 – 2.3)* |  | 42 (8.9) | 3.4 (2.5 – 4.6)* |
| **CME** | 2,312 (1.3) |  | 1,070 (0.9) |  | 1,216 (2.1) |  | 12 (1.1) | 1.1 (0.6 – 2) |  | 14 (1.6) | 1.7 (0.98 – 2.8) |
| **Corneal transplant^4^** | 142 (0.08%) |  | 48 (0.04) |  | 92 (0.16) |  | 0 | – |  | 2 (0.23) | 4.8 (1.1 – 20.5) |
| **Trabeculectomy or glaucoma tube** | 139 (0.08%) |  | 50 (0.04) |  | 86 (0.15) |  | 0 | – |  | 3 (0.35) | 8.2 (2.3 – 29.2) |

Abbreviations: PEX = pseudoexfoliation, copath = copathologies, RR = relative risk, IOL = intraocular lens, IOP = intraocular pressure, CME = cystoid macular edema

* Statistically significant

1 compared to -ve PEX -ve copath in a multivariable model adjusted for age, sex, diabetes status, small pupil, and advanced cataract.

2 In an additional multivariable model adjusted for above factors as well as zonular dialysis and capsular tension ring use, RR for +ve PEX -ve copath compared to -ve PEX -ve copath = 3.8, 95% CI = 2.3 – 6.4.

3 IOP spike is defined as first recorded postoperative IOP within 3 months > 21 mmHg

4 includes penetrating and endothelial keratoplasty

**Supplemental Table 3**. Visual acuity data in eyes with and without pseudoexfoliation

| **VA (logMAR)** | **N** |  | **Overall** |  | **-ve PEX -ve copath** |  | **-ve PEX +ve copath** |  | **+ve PEX -ve copath** | |  | **+ve PEX +ve copath** | |
| --- | --- | --- | --- | --- | --- | --- | --- | --- | --- | --- | --- | --- | --- |
|  |  |  | **Mean ± SD / N (%)** |  | **Mean ± SD / N (%)** |  | **Mean ± SD / N (%)** |  | **Mean ± SD / N (%)** | **Adjusted difference / RR (95% CI)^1^** |  | **Mean ± SD / N (%)** | **Adjusted difference / RR (95% CI)^1^** |
| **Preoperative** | 171,542 |  | 0.64 ± 0.54 |  | 0.59 ± 0.50 |  | 0.74 ± 0.60 |  | 0.74 ± 0.61 | 0.08 (0.05 – 0.11)* |  | 0.93 ± 0.72 | 0.2 (0.17 – 0.24)* |
| **Postoperative** |  |  |  |  |  |  |  |  |  |  |  |  |  |
| **0 – 4 weeks** | 64,201 |  | 0.31 ± 0.40 |  | 0.25 ± 0.32 |  | 0.42 ± 0.48 |  | 0.39 ± 0.48 | 0.10 (0.06 – 0.13)* |  | 0.56 ± 0.61 | 0.25 (0.21 – 0.29)* |
| **4 – 12 weeks** | 91,046 |  | 0.19 ± 0.32 |  | 0.14 ± 0.25 |  | 0.29 ± 0.40 |  | 0.21 ± 0.38 | 0.04 (0.02 – 0.07)* |  | 0.38 ± 0.52 | 0.2 (0.17 – 0.23)* |
| **12 – 24 weeks** | 43,681 |  | 0.23 ± 0.35 |  | 0.17 ± 0.27 |  | 0.33 ± 0.44 |  | 0.25 ± 0.33 | 0.03 (-0.01 – 0.07) |  | 0.46 ± 0.54 | 0.23 (0.19 – 0.28)* |
| **Postoperative VA ≤ 0.3 logMAR^2^** | 91,046 |  | 76,674 (84%) |  | 53,661 (89.1%) |  | 22,219 (74.6%) |  | 486 (85.4%) | 0.99 (0.95 – 1.02) |  | 308 (67.8%) | 0.79 (0.75 – 0.84)* |
| **VA gain ≥ 0.3 logMAR^2^** | 90,872 |  | 56,679 (62%) |  | 38,674 (64.4%) |  | 17,349 (58.3%) |  | 383 (67.3%) | 1.05 (0.99 – 1.11) |  | 273 (60.5%) | 0.93 (0.87 – 1.0) |

Abbreviations: PEX = pseudoexfoliation, copath = copathologies, RR = relative risk, VA = visual acuity, logMAR = logarithm of the minimum angle of resolution

* Statistically significant

1. compared to -ve PEX -ve copath in a multivariable model adjusted for age, sex, diabetes status, small pupil, and advanced cataract

2. at 4 – 12 weeks
